# Application of the case-mix index and length of stay for hospital waste management comparison: introduction of a new adjusted metric

**DOI:** 10.3389/fpubh.2025.1623725

**Published:** 2025-11-12

**Authors:** Adam Kaposi, Attila Nagy, Gabriella Gomori, Denes Kocsis

**Affiliations:** 1 Department of Hospital Hygiene, University of Debrecen Clinical Centre, Debrecen, Hungary; 2 Coordinating Centre for Epidemiology, University of Debrecen Clinical Centre, Debrecen, Hungary; 3 Department of Health Informatics, Faculty of Health Sciences, University of Debrecen, Debrecen, Hungary; 4 Department of Environmental Engineering, Faculty of Engineering, University of Debrecen, Debrecen, Hungary

**Keywords:** case-mix index, length of stay, healthcare waste management, sustainability, waste generation rate, hazardous waste

## Abstract

**Introduction:**

Hazardous healthcare waste (HHCW) presents escalating environmental and operational challenges, yet traditional indicators such as waste generation rate (kg/bed/day) fail to account for patient complexity or care intensity, leading to biased institutional comparisons. Despite various previous normalization attempts, no validated framework has yet integrated clinical and operational heterogeneity into a single benchmarking metric. This study introduces and validates the Complexity-Adjusted Waste Index (CAWI), a novel metric that integrates the Case-Mix Index (CMI) and Length of Stay (LOS) to normalize waste generation across hospitals with heterogeneous clinical profiles.

**Methods:**

Using national data from 94 inpatient institutions in Hungary (2017–2021), CAWI was calculated and compared with conventional HHCW generation rates through Spearman correlation, Fisher’s *Z*-tests, and robust regression models.

**Results:**

Results show that higher CMI correlates with increased HHCW (*r* = 0.49, *p* < 0.001), while shorter LOS is associated with higher daily waste intensity (*r* = −0.67, *p* < 0.001). CAWI demonstrated reduced statistical dispersion (SD = 0.15 vs. 0.27) and stronger correlations with key institutional variables, including number of ICU-patients (*r* = 0.78 vs. 0.67) and number of inpatients (*r* = 0.71 vs. 0.54), with significantly lower model error terms.

**Discussion:**

By explicitly combining patient complexity and treatment intensity into a transferable normalization framework, CAWI advances current benchmarking approaches both theoretically and methodologically. The CAWI framework offers a statistically robust and scalable solution for complexity-sensitive benchmarking, enabling more accurate cross-institutional comparisons and supporting targeted waste reduction strategies aligned with circular economy principles.

## 1 Introduction

The waste generation of healthcare institutions has been continuously increasing in recent years, with a global annual rise of 2–3% (1, 2). This growth poses significant environmental and economic challenges, as the management of healthcare waste (HCW) requires specialized procedures and entails substantial costs (3–5).

HCW presents a critical environmental and public health issue across both high-income and low- and middle-income countries (LMICs), with marked disparities in waste management infrastructures, regulations, and enforcement mechanisms (6, 7). In developed nations, regulatory frameworks - such as the EU Waste Framework Directive or the World Health Organization (WHO) guidelines - offer robust standards for waste segregation, transportation, treatment, and disposal (8, 9). However, compliance gaps and rising volumes due to aging populations and medical technology expansion remain persistent challenges (10, 11). In contrast, many LMICs face acute constraints, including inadequate policy implementation, infrastructural limitations, and limited public awareness, leading to unsafe handling and uncontrolled dumping of infectious or hazardous healthcare waste (HHCW) (12, 13).

HCW is typically categorized into general (non-hazardous) and hazardous components, the latter representing about 15–25% of total waste generated by healthcare facilities (9, 14). HHCW includes sharps, pathological waste, infectious materials, pharmaceutical and chemical waste, and radioactive items (9). The WHO provides clear definitions and classifications, emphasizing the critical need for safe handling to mitigate exposure risks for healthcare workers, waste handlers, and communities (3, 9).

Improper management of HHCW entails serious health and ecological risks. Exposure to pathogens, toxic chemicals, and radioactive substances can cause infections, injuries, and chronic diseases (15, 16).

Recent studies have emphasized that the COVID-19 pandemic significantly exacerbated existing problems. The volume of infectious waste spiked due to increased personal protective equipment (PPE) use, testing materials, and patient care disposables (17, 18). To address these challenges, researchers are increasingly advocating circular economy strategies, including green procurement, waste minimization at source, and reuse of safe materials (17, 19, 20). European healthcare institutions, for example, have successfully adopted reusable protective equipment, advanced sterilization and waste-to-resource technologies, achieving significant reductions in HHCW (4, 5).

The amount of hospital waste can vary significantly depending on the type of institution, the composition of the treated patient population, the nature of medical interventions, and the treatment protocols applied (21, 22). Comparing waste generation among healthcare institutions is therefore a complex task, influenced by multiple factors.

The most commonly used metric for comparing hospital waste generation is kg/bed/day (9, 23).

Despite the widespread use of the kg/bed/day indicator, it introduces significant distortions, as it does not consider the complexity of treated patients, the intensity of care, or the duration of hospital stay.

Although numerous studies have examined the determinants of hospital waste generation, most have relied on partial or single-factor normalization approaches—for example, adjusting by bed numbers, patient days, or hospital size—without accounting for the clinical and operational complexity underlying waste generation patterns. Consequently, no statistically validated, complexity-sensitive metric currently exists that integrates key influencing factors—such as patient complexity and treatment duration—into a single, unified, and transferable framework.

The Case-Mix Index (CMI) is a globally recognized indicator that measures the average resource intensity and complexity of care required by a hospital’s patient population (24, 25).

The CMI reflects patient complexity: the higher the value, the more severe and resource-intensive the cases treated by the hospital (26, 27).

The Length of Stay (LOS) plays a crucial role in waste generation, yet it is often overlooked.

Several studies have documented the association that shorter LOS, characteristic of acute care institutions with high patient turnover, is associated with higher daily waste generation due to increased intensity of care, frequent use of single-use diagnostic materials, and PPE (28, 29). In contrast, hospitals characterized by longer LOS typically provide less intensive daily interventions, thus resulting in comparatively lower daily waste generation rates (30, 31). Based on this evidence, we consider LOS a critical proxy for institutional care intensity and profile. Since shorter stays inherently correlate with more resource-intensive care and higher per-day waste, disregarding LOS may distort hospital-level waste comparisons. Therefore, incorporating LOS into waste metrics is essential for adjusting for these structural differences and ensuring a fairer, complexity-sensitive assessment.

Despite the evident need for multidimensional, data-driven indicators capable of capturing the operational and clinical heterogeneity of healthcare institutions, previous normalization attempts remain limited in scope, lacking both statistical rigor and cross-system applicability. This highlights a clear research gap in establishing a robust, empirically validated, complexity-adjusted methodology that can support equitable and internationally comparable assessments of hospital waste practices.

To address this gap, we introduce the *Complexity-Adjusted Waste Index (CAWI)* - novel, statistically validated metric that integrates the CMI and LOS to normalize HHCW generation across institutions with differing clinical profiles. This is the first framework to jointly incorporate these operational dimensions, enabling fair and context-sensitive benchmarking of hospital waste generation.

The CAWI enables:

- More precise benchmarking of hospital waste management efficiency.
- Identification of departments or institutions generating excessive waste relative to their complexity and patient stay duration.
- The development of targeted waste reduction strategies, such as optimizing material use and reducing reliance on single-use items.

This study develops and statistically validates CAWI as an empirically grounded framework that addresses the long-standing absence of complexity-sensitive normalization in hospital waste benchmarking. By filling this methodological gap, CAWI establishes a new analytical foundation that enhances accuracy, fairness, and comparability in waste assessment Beyond its theoretical contribution, CAWI provides a practical decision-support tool for hospital managers to benchmark performance, identify inefficiencies, and design targeted waste reduction strategies.

The subsequent Literature Review section synthesizes existing measurement approaches and their evolution, positioning CAWI within the broader context of healthcare waste benchmarking models.

## 2 Literature review: evolution of hospital waste performance metrics

This section reviews the evolution of hospital waste performance metrics, summarizing the classical generation indicators, the key influencing factors, and prior normalization attempts that laid the groundwork for complexity-sensitive approaches such as CAWI.

### 2.1 Classical waste generation indicators

Early investigations employed simple unit-based indicators—such as kilograms of waste per bed, per patient, or per day—to quantify and compare hospital waste generation. These classical unit generation rates have been widely applied due to their simplicity and data availability, yet they fail to reflect the operational and clinical heterogeneity of healthcare institutions.

Several simpler indicators have been historically employed to benchmark hospital waste generation:

- kg/day – This indicator represents the total daily waste generation of a healthcare institution. While easy to compute and useful for capturing gross scale, it does not account for institutional size (e.g., number of beds) or patient turnover, which limits its comparability across hospitals (32).
- kg/year – Suitable for long-term trend analysis and annual reporting, this metric aggregates total waste over a year. However, it fails to differentiate between different types of healthcare services, or to adjust for variations in patient throughput or case-mix (33).
- kg/patient – Accounts for patient flow more accurately by relating total waste to the number of treated patients, but it does not distinguish between patients with different medical needs, treatment types, or resource requirements, thereby limiting its comparability across healthcare facilities (34).

Following these, the most commonly used unit generation rate indicator is kg/bed/day, which divides daily waste by the number of beds. This metric offers a familiar unit for hospital benchmarking, and its advantages include ease of use and wide reporting availability (9). However, its limitations must be highlighted: it assumes homogeneity in bed usage and case complexity, ignores differences in patient stay lengths, care intensity, patient mix, and treatment protocols. As shown by Debere et al. (35), the HCW generation rate (in kg/bed/day or kg/patient/day) is not a homogeneous or practical indicator for benchmarking, as it fails to account for hospital size, specialization, technical capacity or quality of care. Hospitals with higher levels of specialization or resource-intensive procedures will naturally generate more waste—even if their waste management practices are efficient (36). Therefore, direct comparisons of waste generation rates across institutions are misleading, and an adjustment method is required to ensure fair and meaningful benchmarking.

### 2.2 Influencing factors of hospital waste generation

Previous studies have demonstrated that university-affiliated institutions engaged in teaching and research activities tend to generate more waste due to the higher consumption of materials and equipment (37, 38). Such institutional characteristics inherently distort traditional indicators such as kg/bed/day, since these facilities appear less efficient despite their higher research and procedural intensity rather than poor waste management performance (22).

Furthermore, patient flow—particularly the proportion of intensive care and surgical patients—has been closely linked to the volume of infectious and hazardous waste. Hospitals with larger shares of surgical or ICU-patients inevitably report higher unit generation rates per bed or patient, as these departments require more consumables, sterilization materials, and single-use protective items. Without adjustment for these clinical factors, comparisons between hospitals of different profiles become misleading (31, 33, 37).

Healthcare-associated infections (HAIs), especially those caused by multidrug-resistant bacteria, significantly increase the use of single-use protective equipment and isolation materials, thereby contributing to higher waste generation (31, 39).

Additionally, legal regulations and definitions applied in different countries are not standardized, making international comparisons challenging (9, 40). Differences in waste classification thresholds and segregation policies further compound these discrepancies, leading to inconsistencies in reported kg/bed/day or kg/patient/day values across regions (23).

Overall, these structural, clinical, and regulatory variations introduce systematic bias into conventional waste generation indicators, emphasizing the need for normalization methods that correct for hospital complexity, service mix, and infection burden (21).

### 2.3 Previous normalization attempts

Efforts to normalize healthcare waste indicators have sought to correct for institutional or clinical differences influencing waste generation, yet most approaches have remained limited in scope and statistical validation.

Xin (36) demonstrated a positive association between the CMI and HCW generation, highlighting that institutions treating more complex cases tend to produce higher volumes of waste, thereby supporting the need for complexity-adjusted performance metrics in waste management. However, the study’s statistical analysis did not confirm this relationship with sufficient significance, and the author emphasized that additional, unmeasured factors likely influence waste generation, warranting further research across more diverse hospital settings.

Eker and Bilgili (30) applied a statistical regression model in Turkish healthcare facilities, identifying bed capacity, patient admissions, and inpatient days (LOS) as significant predictors of healthcare waste generation. Their model confirmed that waste output cannot be reliably assessed by single-dimension indicators such as kg/bed/day alone.

Minoglou et al. (21) conducted a meta-analysis across global datasets and found that hospital type, economic development, and environmental conditions collectively explained most of the variation in generation rates. These results supported a shift toward multi-factor, context-adjusted modeling. However, while their synthesis identified key determinants of waste generation, it did not establish a formal statistical correction or normalization framework capable of adjusting raw indicators for these confounding variables.

Studies in Greek public hospitals reported that LOS, bed occupancy, and throughput metrics explained most of the variance in infectious waste and its management costs, underscoring the importance of incorporating hospital operational intensity into benchmarking frameworks (32).

These studies collectively demonstrate that multi-variable adjustment improves explanatory power compared with static per-bed or per-patient ratios. However, none of them developed a standardized or statistically validated correction model capable of transforming these predictors into a transferable normalization index, leaving cross-institutional comparability unresolved.

Emerging data-driven approaches have further enhanced predictive accuracy by integrating features such as patient inflow, ICU ratio, and procedural intensity (41). Recent machine-learning models applied to healthcare waste prediction achieved explained variances above 0.9, demonstrating superior short-term performance compared with traditional regression; however, these models remain exploratory and lack transparent, generalizable formulas that could serve as operational benchmarking tools (42).

Taken together, contemporary evidence supports replacing static single-unit generation rates with multi-factor, complexity-aware models that explicitly incorporate institutional workload, clinical intensity, and LOS. Yet, despite these advances, no statistically validated or universally applicable normalization framework currently exists—thereby justifying the development of the CAWI model as the first empirically grounded, complexity-adjusted benchmarking tool for healthcare waste management.

This cumulative progression from single-dimension metrics to multi-factor, complexity-adjusted models forms the conceptual and methodological foundation from which the CAWI framework has been developed.

## 3 Methods

### 3.1 Study area, and period

This study analyzed HHCW generation in 94 inpatient healthcare institutions in Hungary over a five-year period (2017–2021), collectively covering 94.4% of the country’s inpatient care capacity. These institutions performed comprehensive medical services across the full spectrum of clinical specialties during the study period, including both acute and chronic care. The sample encompasses a wide range of hospital types - including national, university-affiliated, county, city, specialist, and other general hospitals - and is geographically distributed across Hungary’s seven administrative regions (31).

While detailed institutional characteristics - such as hospital type, bed capacity, and regional location - were comprehensively described in our previous work (31), no patient-level demographic data were included in that dataset. Nevertheless, to provide contextual background, Hungary has a population of 9.58 million (2024), a territory of 93,012 km^2^, and a gross domestic product (GDP) per capita of €28,700 (2024) (43).

Only institutions with available CMI data were included in this analysis. This effectively limited the dataset to Hungary’s 94 largest inpatient hospitals, as CMI data are generally not reported for smaller institutions - typically those with fewer than 30 inpatient beds. This selection criterion enables more accurate normalization of waste generation by incorporating patient complexity while preserving the representativeness of the national inpatient care landscape.

Importantly, no pre-selection was made based on institutional type or region, ensuring that the analysis includes diverse healthcare settings and service profiles, thereby enhancing the generalizability and robustness of the proposed indicator.

### 3.2 Data sources

For the validation of this indicator, HHCW was selected as the primary category of waste under investigation. The choice of HHCW is driven by its well-established global definition by the WHO and its broad applicability across different healthcare systems worldwide (9). HHCW, as defined by the WHO, including infectious waste, pathological waste, cytotoxic and pharmaceutical waste, chemical waste, radioactive waste, and sharps. This broad classification ensures that the proposed indicator is universally applicable across different healthcare systems, facilitating international benchmarking.

For the study period (2017–2021), the annual quantities of HHCW for each institution were obtained from the online interface of the National Environmental Information System, ensuring standardized and consistent data collection across institutions and years (44). The patient complexity data, represented by the CMI values, and the LOS data were obtained from the annual reports published by the National Health Insurance Fund (45).

To support the comparative evaluation of the two waste indicators, hazardous healthcare waste generation rate (HHCW GR) and CAWI, we included additional control factors with proven relevance to HHCW generation, as established in previous national-level research (31, 33, 37–39). Annual data on the total number of inpatients and the number of intensive care unit (ICU) patients were obtained from the administrative datasets of the National Health Insurance Fund (45). These variables serve as proxies for institutional scale and clinical intensity, both of which influence waste output.

The incidence of healthcare-associated infections caused by multidrug-resistant (MDR) bacteria - expressed as cases per 100,000 inpatient days - was retrieved from the National Nosocomial Surveillance System maintained by the National Public Health and Pharmaceutical Center (46). This indicator reflects infection control burden and correlates with increased use of isolation materials and PPE.

Educational activity was included as a binary variable, serving as an indicator of whether the hospital had an academic function. Such institutions typically generate more waste due to higher diagnostic activity and teaching-related procedures.

### 3.3 Hospital grouping and classification criteria

The institutions were categorized into five distinct groups based on their bed capacity to analyze variability in waste generation according to hospital size and treatment complexity. The classification was established using quintile-based stratification, ensuring that each group contained approximately 20% of the institutions. This approach allows for balanced comparison across hospital sizes while preserving statistical representativeness. The resulting groups were as follows:

- Group 1: small hospitals (30–242 beds).
- Group 2: medium-sized hospitals (253–377 beds).
- Group 3: moderately large hospitals (378–591 beds).
- Group 4: large hospitals (592–1,033 beds).
- Group 5: largest hospitals (1,128–3,513 beds).

### 3.4 Indicator development and mathematical formulation

The CMI is calculated using the following formula:

$$\mathrm{CMI}=\frac{\sum (\text{Relative weight}\times \text{Number of cases})}{\text{Total number of cases}}$$

- Relative Weight refers to the Diagnosis-Related Group (DRG) weight assigned to each patient group, representing the expected resource consumption for their treatment.
- Number of Patients is the count of patients within a specific DRG category.
- Total Number of Patients is the total number of patients treated in the healthcare facility (24, 47).

To account for treatment duration, the LOS was included in the analysis. LOS is defined as the average number of inpatient days per hospital stay and was calculated using the formula:

$$\mathrm{LOS}=\frac{\text{Total inpatient days}}{\text{Total discharges}}$$

To enable more equitable comparisons of hospital waste generation across institutions with varying case complexity and care intensity, we developed a mathematically normalized indicator. The CAWI is defined as:

$$\text{CAWI}=\frac{\text{HHCW}\;\mathrm{GR}\times \mathrm{CMI}}{\mathrm{LOS}}$$

where:

- HHCW GR is the hazardous healthcare waste generation rate in kg/bed/day.
- CMI is the Case-Mix Index reflecting the average clinical complexity and resource intensity of the hospital.
- LOS is the average Length of Stay (in days), used as a proxy for institutional care intensity.

This formulation is based on the rationale that while the traditional waste indicator (kg/bed/day) captures raw waste intensity, it fails to reflect patient complexity; therefore, the CMI is introduced as a multiplier to account for clinical severity and resource use, whereas the average LOS is applied as a divisor to adjust for care intensity, recognizing that shorter hospital stays often correlate with more intensive treatments and higher daily waste output. Thus, the CAWI index captures the expected waste per bed per day, normalized for both clinical complexity and treatment intensity. In mathematical terms, it is a second-order normalized ratio, aiming to express the relative waste performance of an institution independent of structural and clinical heterogeneity. The resulting index is expressed in kg/bed/day^2^, but in practice it functions as a comparative, scale-independent metric.

### 3.5 Data analysis

The statistical analysis started with the Shapiro–Wilk W test to check for normality. Since the data were non-normally distributed, non-parametric methods were applied.

First, an F-test was conducted to compare the variance of HHCW GR and CAWI. The relationship between CMI, LOS, and HHCW GR was examined using Spearman’s correlation, given the non-normal data distribution. The statistical analysis included several steps to compare HHCW GR and the proposed CAWI, considering control variables: ICU-patient, incidence of MDR bacterial infections (per 100,000 inpatient days), number of inpatients, and educational activity. Next, a Spearman correlation analysis was performed to assess the association between both waste metrics (HHCW GR and CAWI) and the control variables. Fisher’s Z-test was applied to compare the correlation coefficients, determining if CAWI better accounts for confounding factors. Finally, robust regression analysis evaluated the impact of control variables on HHCW GR and CAWI, assessing whether CAWI provides a more stable and accurate measure of hospital waste generation.

In our analysis, a *p*-value of less than 0.05 was considered significant. Intercooled Stata v17 was used for the analysis.

## 4 Results

The analysis of hospital waste generation shows significant variability across institutions (Table 1). Smaller hospitals (Group 1) had the lowest HHCW GR (0.26 kg/bed/day), while the largest hospitals (Group 5) reported the highest (0.51 kg/bed/day).

**Table 1:** Comparison of hazardous healthcare waste generation rate (HHCW GR), patient complexity (Case-Mix Index, CMI), and Length of Stay (LOS) across hospital size groups.

Hospital groups	HHCW GR [kg/bed/day] (SD*)	CMI (SD*)	LOS (SD*)	CAWI [GR x CMI/LOS] (SD*)
Group 1 (30–242 beds)	0.26 (0.26)	0.76 (0.48)	17.23 (9.60)	0.05 (0.14)
Group 2 (253–377 beds)	0.33 (0.37)	1.05 (0.81)	15.69 (13.65)	0.10 (0.28)
Group 3 (378–591 beds)	0.30 (0.22)	1.04 (0.33)	14.94 (20.75)	0.04 (0.03)
Group 4 (592–1,033 beds)	0.32 (0.15)	1.18 (0.36)	9.07 (3.72)	0.05 (0.03)
Group 5 (1,128–3,513 beds)	0.51 (0.23)	1.29 (0.18)	6.87 (1.38)	0.12 (0.09)
	Total SD*: 0.27			Total SD*: 0.15

*SD, Standard deviation.

Examining the CMI and LOS values, Group 1 (30–242 beds) had the lowest complexity (CMI = 0.76; SD = 0.48) and the longest LOS (17.23 days, SD = 9.60), whereas Group 5 (1,128–3,513 beds) had the highest complexity (CMI = 1.29; SD = 0.18) and the shortest LOS (6.87 days, SD = 1.38). Intermediate hospital groups (Groups 2–4) exhibited CMI values between 1.04 and 1.18, and LOS values between 9.07 and 15.69 days. Group 3 (378–591 beds) showed relatively low dispersion for CMI (SD = 0.33) and CAWI (SD = 0.03), indicating more homogeneous characteristics in this group.

The highest CAWI value was observed in Group 5 (0.12, SD = 0.09), followed by Group 2 (0.10, SD = 0.28). Group 3 recorded the lowest CAWI (0.04, SD = 0.03), while Groups 1 and 4 had intermediate values (0.05 in both cases), but with different levels of variability (SD = 0.14 and 0.03, respectively). Larger tertiary hospitals (Group 5) exhibited the highest CAWI values together with shorter LOS, suggesting that the index more accurately reflects high-intensity care settings.

A direct comparison of the two indicators shows that HHCW GR had a higher standard deviation (total SD = 0.27) than CAWI (total SD = 0.15). The difference in variances between the two indicators was statistically significant (*F*-test: *p* < 0.001). The variability of the traditional generation rate was markedly higher than that of CAWI, indicating that CAWI yields more homogeneous benchmarking across institutions with different clinical profiles.

**Figure 1:**
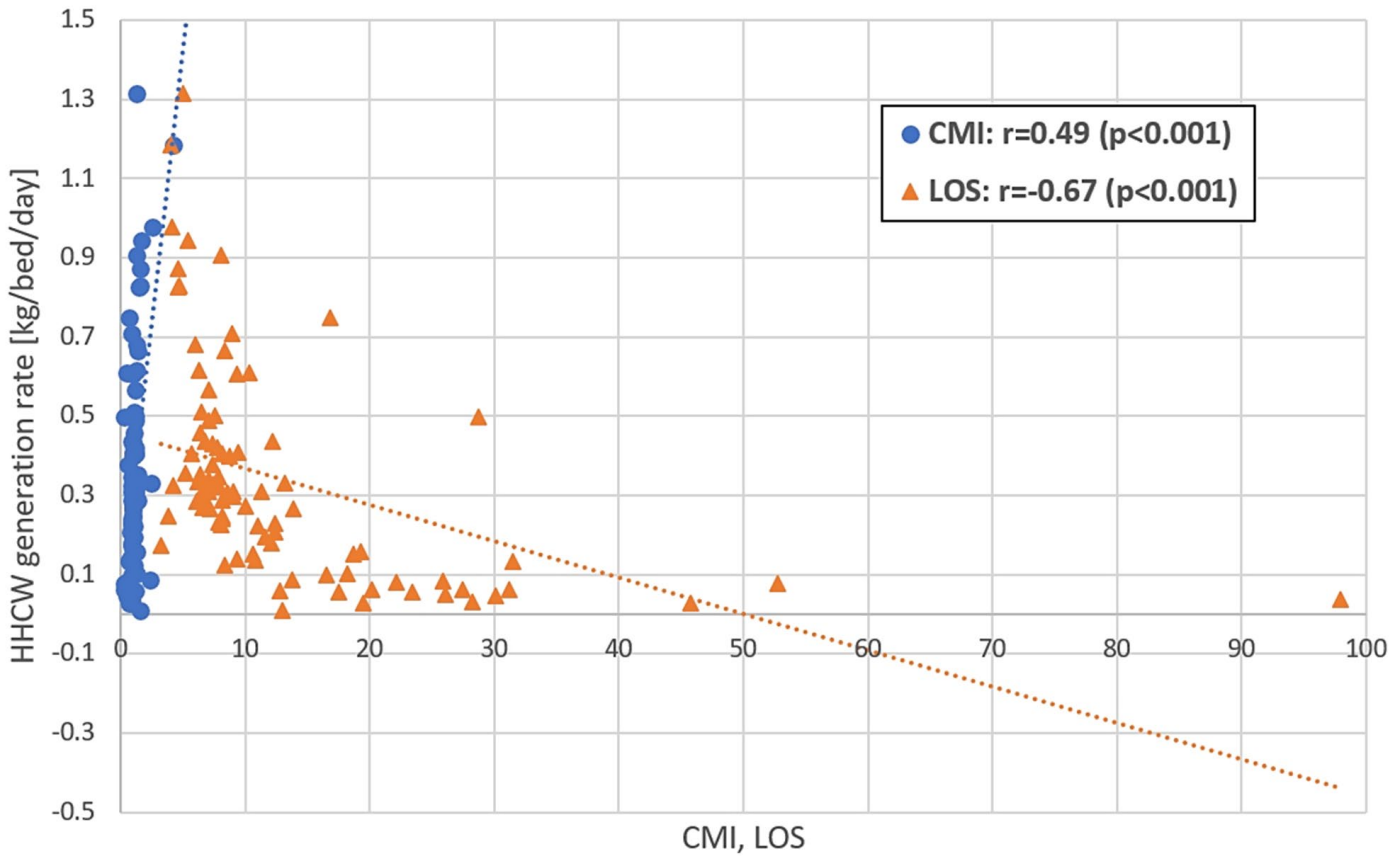
Spearman’s correlation between Case-Mix Index (CMI), Length of Stay (LOS), and hazardous healthcare waste generation rate (HHCW GR).

The Spearman correlation analysis confirmed statistically significant associations between CMI, LOS, and HHCW GR (Figure 1). A moderate positive correlation was found between CMI and HHCW GR (*r* = 0.49, *p* < 0.001). LOS showed a strong negative correlation with HHCW GR (*r* = −0.67, p < 0.001). These correlations were consistent across the full institutional dataset (*n* = 94), with no major deviation observed in subgroup distributions.

The correlation analysis with external control variables revealed that CAWI showed stronger associations than HHCW GR across most examined factors (Table 2). The correlation coefficient between CAWI and the number of ICU-patients was *r* = 0.78 (*p* < 0.001), compared to *r* = 0.67 (*p* < 0.001) for HHCW GR. For the incidence of MDR bacterial infections, the correlation was *r* = 0.62 (*p* < 0.001) for CAWI and *r* = 0.57 (*p* < 0.001) for HHCW GR. The number of inpatients was also more strongly correlated with CAWI (*r* = 0.71, *p* < 0.001) than with HHCW GR (*r* = 0.54, *p* < 0.001). Fisher’s *Z*-test indicated statistically significant differences in correlation coefficients for ICU-patients and number of inpatients (*p* < 0.001 in both cases). The difference for MDR bacterial infections was not statistically significant (*p* = 0.216).

**Table 2:** Spearman’s correlation between waste generation indicators - hazardous healthcare waste generation rate (HHCW GR) and Complexity-Adjusted Waste Index (CAWI) - and institutional control factors.

Control factors	HHCW GR [kg/bed/day]		CAWI [GR x CMI/LOS]		*Z*-score *p*-values
	*r*	*p*-values	*r*	*p*-values	
Number of ICU-patients	0.67	<0.001	0.78	<0.001	<0.001
Incidence of HAIs caused by MDR bacteria (per 100,000 inpatient days)	0.57	<0.001	0.62	<0.001	0.216
Number of inpatients	0.54	<0.001	0.71	<0.001	<0.001

ICU, intensive care unit; HAI, healthcare-associated infection; MDR, multidrug-resistan.

The robust regression analysis further supported the superior statistical performance of the CAWI model over the HHCW GR model across all examined control variables (Table 3). Notably, standard errors were consistently lower when CAWI was applied: 2.27 × 10^−6^ versus 1.40 × 10^−5^ for ICU-patients, 3.65 × 10^−5^ versus 2.19 × 10^−4^ for the incidence of HAIs caused by MDR bacteria, 2.22 × 10^−3^ versus 1.36 × 10^−2^ for educational activity, and 7.03 × 10^−8^ versus 4.30 × 10^−7^ for the number of inpatients.

**Table 3:** Robust regression analysis of hazardous healthcare waste generation rate (HHCW GR) and Complexity-Adjusted Waste Index (CAWI) with institutional control variables.

Control factors	HHCW GR [kg/bed/day]			CAWI [GR x CMI/LOS]		
	Coefficient	*p*-values	SE	Coefficient	*p*-values	SE
Number of ICU-patients	1.67 × 10^−4^	<0.001	1.40 × 10^−5^	4.23 × 10^−5^	<0.001	2.27 × 10^−6^
Incidence of HAIs caused by MDR bacteria (per 100,000 inpatient days)	1.45 × 10^−3^	<0.001	2.19 × 10^−4^	1.38 × 10^−4^	<0.001	3.65 × 10^−5^
Educational activity*	1.47 × 10^−1^	0.005	1.36 × 10^−2^	2.01 × 10^−2^	0.004	2.22 × 10^−3^
Number of inpatients	−1.23 × 10^−6^	<0.001	4.30 × 10^−7^	−2.00 × 10^−7^	<0.001	7.03 × 10^−8^

*Conducts accredited educational activities.

ICU, intensive care unit; HAI, healthcare-associated infection; MDR, multidrug-resistant.

**Figure 2:**
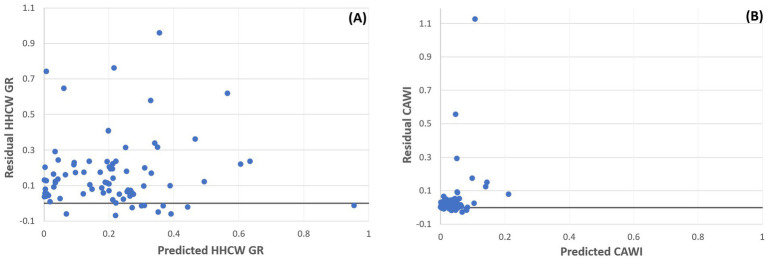
Residual plots of robust regression models comparing hazardous healthcare waste generation rate (HHCW GR) and the Complexity-Adjusted Waste Index (CAWI) across institutional control variables. **(A)** Residuals of the HHCW GR model. **(B)** Residuals of the CAWI model.

In line with these findings, the regression coefficients were also systematically smaller under the CAWI model, suggesting reduced sensitivity to variability: 4.23 × 10^−5^ compared to 1.67 × 10^−4^ for ICU-patients, 1.38 × 10^−4^ versus 1.45 × 10^−3^ for MDR infections, 2.01 × 10^−2^ versus 1.47 × 10^−1^ for educational activity, and −2.00 × 10^−7^ versus −1.23 × 10^−6^ for the number of inpatients. All associations remained statistically significant (*p* < 0.05), reinforcing the enhanced robustness and interpretability of the CAWI model. To visualize model performance differences, residual plots were generated for both regression models (Figure 2). The traditional HHCW GR model exhibited wider and more heteroscedastic residual dispersion, whereas the CAWI model showed a more compact, symmetric distribution of residuals around zero, indicating improved model fit and reduced unexplained variability.

Taken together, these results indicate that CAWI provides a statistically more reliable and context-sensitive representation of hospital waste generation than the traditional per-bed rate, as it reflects variability primarily driven by clinical complexity and patient turnover rather than random institutional factors.

## 5 Discussion

The findings of this study confirm that hospital waste generation varies substantially across institutions, with higher HHCW GR values typically associated with larger hospital sizes, aligning with previously documented trends in the literature (31, 32).

Our findings regarding the higher HHCW GRs in larger institutions with higher patient complexity and shorter LOS align well with previous research highlighting the increased resource intensity in acute-care and specialized institutions (30, 31, 36). The significant inverse relationship between LOS and daily waste generation is also consistent with earlier studies indicating that shorter hospital stays intensify the use of single-use materials and protective equipment, particularly in high-risk settings such as ICUs (28, 29).

Since the dataset includes nearly all hospitals in the country without pre-selection, the wide range of patient complexity levels contributes to the observed differences in CAWI.

As shown in the Results, CAWI integrates CMI and LOS, reducing statistical dispersion (SD = 0.15 vs. 0.27) and improving comparability across hospitals. The strong correlation patterns between CMI, LOS, and HHCW GR (*r* = 0.49 and −0.67, *p* < 0.001) confirm that shorter hospital stays are associated with increased waste intensity (28).

The improved correlation of CAWI with control variables—particularly ICU-patient numbers (*r* = 0.78 vs. 0.67), MDR infections (*r* = 0.62 vs. 0.57), and inpatient volume (*r* = 0.71 vs. 0.54)—supports the claim that CAWI better captures institutional factors that genuinely influence hazardous waste output.

Regression results further confirmed the robustness of CAWI, with consistently lower standard errors and smaller coefficients across all models.

Beyond its statistical robustness, the CAWI metric carries direct operational value for hospital management. Because it integrates patient complexity and treatment intensity, CAWI allows institutions to compare waste generation on a more equitable basis and to identify departments or service lines that generate disproportionately high volumes. Such information can guide targeted waste minimization and procurement strategies in high-intensity units where single-use protective materials dominate hazardous waste. This operational use extends the indicator’s role from mere benchmarking toward practical decision support. Recent advances in multi-criteria and fuzzy decision-support systems for sustainable medical waste management highlight how such tools could complement CAWI by prioritizing interventions within its normalized benchmarking framework (48). The ability to pinpoint high-impact areas enables resource reallocation toward prevention measures, training, and the adoption of reusable or reprocessable materials without compromising infection control.

Building on this operational foundation, future applications could integrate CAWI into digital hospital management and sustainability systems. Embedding the index within electronic reporting tools, environmental data platforms, or AI-driven decision-support modules would enable real-time assessment of waste generation in relation to patient complexity and care intensity, in line with emerging AI-based circular-economy waste-management frameworks demonstrated in recent studies (49). Such integration would enhance analytical precision and support proactive interventions where waste pressures are highest. Moreover, connecting CAWI with circular economy–oriented digital instruments - such as life-cycle assessment modules or procurement tracking systems - could provide hospitals with data-driven feedback loops for improving material efficiency and closing resource cycles. These developments would position CAWI as a next-generation tool that bridges environmental performance monitoring with intelligent, circular healthcare management.

At the policy level, CAWI offers a standardized framework that could complement existing reporting systems based solely on mass-based indicators. Integrating CAWI into national or regional benchmarking schemes would enhance comparability between hospitals with differing case mixes and promote evidence-based performance evaluation. Recent reviews also emphasize the need to embed environmental sustainability indicators into hospital performance management systems (50). Over time, this may contribute to the development of context-sensitive targets for waste reduction and sustainability reporting in the healthcare sector, aligning institutional management practices with broader environmental and public health goals.

CAWI provides a more nuanced understanding of HHCW generation than the conventional kg/bed/day indicator. It therefore offers a solid evidence base for identifying high-intensity, waste-producing services and for prioritizing circular economy interventions - such as source reduction, reuse, and optimized procurement - where they yield the greatest impact. As highlighted in previous studies, healthcare systems that successfully integrated these approaches, including the adoption of reusable PPE and advanced sterilization methods, have achieved substantial reductions in HHCW (4, 5, 17, 19, 20). In this context, CAWI can support data-driven prioritization of sustainability efforts by highlighting operational areas with disproportionate waste output. Taken together, these results position CAWI as a reliable and equitable indicator for benchmarking waste performance and guiding hospital-level waste management decisions.

Despite its strengths, our study has some limitations that should be considered. Firstly, the analysis focused exclusively on HHCW, and therefore generalizability to other types of HCW remains to be validated. Additionally, our study is based on data from Hungarian inpatient institutions, and although our approach is broadly applicable, specific local conditions and healthcare practices might limit immediate international generalizability. Future research should aim to apply and validate the CAWI metric across different healthcare settings and waste types internationally, enhancing its robustness and applicability as a universal indicator.

Moreover, several data-related and scalability limitations should be acknowledged. The CAWI model relies on aggregated institutional-level data, which, while suitable for national benchmarking, may mask intra-hospital variability. The lack of patient-level or procedure-specific data may limit the precision of complexity adjustment, and variations in diagnostic coding accuracy could introduce minor systematic bias in CMI values.

Regarding scalability, CAWI requires standardized CMI and LOS datasets, typically available in high-income settings but often limited in low- and middle-income countries. Therefore, the application of CAWI in low-resource contexts may be constrained by data availability and inconsistent waste reporting systems, warranting further validation in such environments.

## 6 Conclusion

This study provides substantial evidence that conventional waste metrics, such as kg/bed/day, do not adequately capture the complexity and variability of HHCW generation across diverse institutions. The newly developed and validated CAWI, integrating CMI and LOS, offers a statistically robust alternative that significantly enhances comparability and fairness among healthcare institutions. By reducing variance and better accounting for confounding factors such as patient complexity and treatment duration, CAWI represents a considerable methodological advancement in healthcare waste management.

From a practical standpoint, healthcare managers and policymakers can directly leverage CAWI as a powerful tool to pinpoint departments or hospitals generating disproportionately high HHCW relative to their patient complexity profile. This capability enables targeted, resource-efficient interventions and fosters the development of precise, evidence-based sustainability strategies at the institutional and regional levels.

In conclusion, the introduction and validation of CAWI mark an important step forward in healthcare waste management research. By precisely adjusting for patient complexity and treatment duration, CAWI not only facilitates fairer benchmarking and more accurate waste assessments but also contributes directly to broader sustainability and circular economy initiatives within healthcare systems. Future developments may further enhance CAWI’s value through integration with digital hospital information systems, AI-supported monitoring, and life-cycle–based decision-support tools, strengthening its role as a bridge between data-driven management and circular healthcare transformation. Further research extending CAWI’s application to diverse healthcare environments will be crucial to fully realize its potential as a global standard in sustainable healthcare waste management.

## Data Availability

The datasets presented in this study can be found in online repositories. The names of the repository/repositories and accession number(s) can be found at: https://data.mendeley.com/preview/vz9954jy99?a=8c93c201-4756-426b-865b-c87a29ba7742.
